# The Story of the Insane from Year to Year

**Published:** 1899-12-02

**Authors:** 


### THE STORY OF THE INSANE FROM YEAR TO YEAR.

Twelfth Series.

### EAST SUSSEX ASYLUM AT HAYWARDS HEATH.

Here during the year 1898 the numbers rose from 845 to 907. There were 198 first admissions, 54 readmissions, 56 recoveries, 27 discharged relieved, and 93 deaths. Calculated on the admissions, the recoveries stand at 25·3 per cent., and the deaths calculated on the average number resident at 10·5. Of the deaths, 43 were caused by cerebral and spinal diseases, 8 by consumption, 6 by bronchitis, 6 by pneumonia, and 1 by pleurisy. The insanity in 22 of the year's admissions was caused by moral causes (domestic trouble, adverse circumstances, religion), in 13 cases by intemperance in drink, in 56 by hereditary influences. The asylum is again full, and attempts have been made to meet the difficulty by boarding out patients in other asylums, but this makeshift was found to be inadequate, and temporary buildings for 100 patients are being erected. In addition to this the detached hospital for infectious diseases is now occupied by ordinary patients. The committee say they "have every reason to be satisfied with the management of the institution by Dr. Saunders and with the conduct of his medical colleagues and the officers generally." The weekly cost of maintenance is 9s. 2½d. per head.

### NORTHAMPTON COUNTY ASYLUM.

In this asylum the numbers have increased from 891 to 918. There were 173 first admissions, 29 readmissions, 37 recoveries, 15 discharged relieved, and 99 deaths. The recoveries stand at 29·1 per cent. of the admissions, and the deaths at 10·9 per cent. of the average number resident. Thirty-four of the deaths were caused by cerebral and spinal diseases, 23 by consumption, and 4 by inflammatory affections of the lungs. Of the year's admissions 13 owed their insanity to such moral causes as mental anxiety, domestic trouble, and religion, 25 to intemperance in drink, and 51 to hereditary influences. As already stated, there were 202 admissions, and Dr. Harding says that of this large number only 30 could be looked on as favourable cases for recovery, while the large majority were hopeless. It is stated that the training classes for nurses and attendants have been most successfully conducted by Dr. Goodliffe and Dr. Crookshank. It is well-known that this is one of the asylums in which is given a three years' course of instruction in all departments of nursing, and a really valuable certificate may be obtained by those willing to devote sufficient time and study to the work. The storekeeper resigned owing to ill-health, and was granted a pension of £40 a year. The weekly rate of maintenance is 7s. 6d. per head.

### THE SOUTH YORKSHIRE ASYLUM AT WADSLEY, SHEFFIELD.

This asylum contained 1,630 patients on January 1st, and at the end of the year the number had risen to 1,634. There were 395 first admissions, 64 readmissions, 204 recoveries, and 183 deaths. The recoveries stand at 44·4 per cent. of the admissions, and the deaths at 11·1 per cent. on the average number resident. Both these percentages are above the average. Cerebral and spinal diseases are responsible for 87 of the deaths, pneumonia for 17, congestion of the lungs for 13, and consumption for 23. The lung diseases seem very numerous, but Dr. Kay does not give any explanation of it. There was no infectious or contagious disease during the year, if we except one case of German measles. Training classes for the attendants and nurses are conducted by Dr. Adair and Dr. Wrangham, and Dr. Kay bears testimony to the increased fitness of the attendants and nurses for their work. We are informed that three attendants and six nurses passed the examination for the nursing certificate of the medico-psychological certificate, and we hope that these nurses will continue their studies and Dr. Kay will institute a higher certificate for them. The asylum would seem to be full, or rather more than full, but neither the visitors nor the medical superintendent makes any suggestion as to what is to be done. The visitors say that "the medical superintendent, assistant medical officers, and officials have carried out their duties to the entire satisfaction of the committee." The weekly cost per head is 9s. 4½d.

